# Editorial: Responsible Digital Health

**DOI:** 10.3389/fdgth.2021.841477

**Published:** 2022-01-21

**Authors:** Naseem Ahmadpour, Geke Ludden, Dorian Peters, Karina Vold

**Affiliations:** ^1^Affective Interactions Lab, School of Architecture, Design and Planning, The University of Sydney, Sydney, NSW, Australia; ^2^Department of Design, Production and Management, University of Twente, Enschede, Netherlands; ^3^Dyson School of Design Engineering, Imperial College London, London, United Kingdom; ^4^Institute for the History and Philosophy of Science and Technology, University of Toronto, Toronto, ON, Canada

**Keywords:** digital health (eHealth), design methodology, ethics, technology - ICT, health, well-being

The growing concern over the ethical implications of digital technology used for health has been amplified by the emergency deployment of technologies in an effort to manage a global pandemic. These events have placed even greater urgency on the need for attention to ethical impacts and value fulfillment, and on the need for advances in responsible digital health research and practice.

Furthermore, given that healthcare practitioners are expected to abide by ethical principles that protect the rights and welfare of their patients, we believe that the technologies functioning as tools and agents of well-being and healthcare provision, should be held to the same account. And indeed, an increasing number of researchers are working to ensure that they are. But in order to make progress toward more responsible practice within digital health, we need more systematic approaches, more research into the ethical implications of digital technology use for health, and more guiding examples of responsible practice in this area. The research article collection described herein responds directly to this need.

For the purposes of selection for this special topic, we considered “Responsible Digital Health” to include any intentional systematic effort designed to increase the likelihood of a digital health technology developed through ethical decision making, being socially responsible and aligned with the values and well-being of those impacted by it.

The papers included reflect a number of angles on the topic and reveal research insights on: issues of **equity** (who gets to be healthy?), the impact of **modality** (the unique promises and risks of particular technologies, such as chatbots) and the need for **process** (including frameworks, guidelines and approaches that can contribute to systematic and replicable best practice).

## Equity—Who Gets to be Healthy?

Digital health often has the potential to particularly serve vulnerable populations, so preventing these technologies from doing harm is both a critical research problem and a moral obligation facing designers and technologists. Protecting and empowering the people who use digital health technologies often requires users' involvement in design, as well as addressing issues of autonomy-support, justice, and equitable access. Faber et al. address these issues in both topic and method in “Attitudes toward health, healthcare, and eHealth of people with a low socioeconomic status: a community-based participatory approach.” Through a participatory approach, the authors explored the attitudes in low Socio-Economic Status (SES) communities toward health, healthcare, and ehealth interventions. Their findings highlight that negative health attitudes are complex and underlined by a range of attitudes like *encumbered* toward health, feeling *disadvantaged* within healthcare, and *hesitance* toward eHealth adoption.

Moreover, there are challenges and opportunities particular to young people with respect to digital health. Wies et al. report on a scoping review that they conducted in order to map the landscape of emerging ethical challenges related to this dually vulnerable population. Their paper “Digital Mental Health for Young People: A Scoping Review of Ethical Promises and Challenges” reveals both the significant promises for youth mental health (e.g., reducing stigma and suffering, while improving access and well-being) as well as the real challenges in delivering on these. They argue that some of the ethical challenges that are raised around the use of digital health devices, such as challenges related to privacy, equality of access, and patient autonomy, may be exacerbated when used by adolescents, as youth are particularly vulnerable and are often below the age of consent for medical treatments.

Similarly, additional reviews within the mental health space provide evidence for both efficacy and for gaps with ethical implications. For example, in a review of the landscape of mobile apps for digital mental health in Spanish, Oñate Muñoz et al. reveal that, while thoughtfully designed apps could hold the key for reducing mental health disparities among Spanish-speakers in the United States, currently available technologies are inadequate.

## Modality—Technology Itself Matters

Digital health technologies encompass the full gamut of modalities, from apps and wearables to data-driven tracking systems, robotic caregivers, telemedicine, Virtual/Augmented Reality (VR/AR), and chatbots. Therefore, research is needed that identifies the ethical implications specific to the use of these different technological approaches for health.

Christoforakos et al. interrogate the impacts of the anthropomorphisation of conversational chatbots on aspects of human experience such as a sense of connectedness with the bot, and implications for human-human interaction. While they found that both regular interaction with the chatbot and a design that facilitates perceptions of anthropomorphism and social presence can foster feelings of social connectedness, they emphasize that the decision to use anthropomorphic technologies should be taken responsibly and may be context dependent.

Vilaza and McCashin provide further insight into chatbot use in their paper, “Is the Automation of Digital Mental Health Ethical? Applying an Ethical Framework to Chatbots for Cognitive Behavior Therapy.” They argue that ethical thinking should be at the core of Artificial Intelligence Cognitive Behavioral Therapy (AI-CBT) design, research and policy, and they also provide a critical overview and framework for assessing the ethical automation of digital mental health therapy.

Roossien et al. shed light on the pros and cons of sensor and intervention technologies for workplace health promotion, in their paper, “Ethics in Design and Implementation of Technologies for Workplace Health Promotion: a Call for Discussion” Through reviewing two cases, they investigated ethical issues, particularly privacy and autonomy, in relation to health technologies for aging workers and draw on challenges of developing and implementing technologies for an aging workforce. The findings reveal how sensors and interventions, so commonly applied to health promotion, can pose significant threats to the autonomy and privacy of workers. To mitigate these consequences, Roossien et al. propose careful consideration of diverse values and perceptions, and to situate those within the responsibilities of workers and employers at the workplace.

Finally, van Lotringen et al. target the affordances and limitations of text. Their paper, “Responsible Relations: A Systematic Scoping Review of the Therapeutic Alliance in Text-Based Digital Psychotherapy” investigates whether important qualities of the therapist-client relationship can be effectively preserved within the constraints of text-only conversational environments.

## Process—Systematic, Rigorous, and Replicable

To create digital health responsibly, we need evidence-based principles, methods, and processes for anticipating and addressing the ethical impacts that technologies have on individuals and society. These often include impacts on core values and rights, such as well-being, autonomy, privacy, and justice.

For example, in “Designing Informed Consent for Digital Health Research: Applying the Digital Health Checklist and Readability Tools to Support Accessible Content,” authors Nebeker et al. provide practical guidance and tools for improving informed consent for digital health research. The work of Vilaza and McCashin, mentioned above, also includes an ethical framework for assessing the use of automation for the delivery of online cognitive behavioral therapy.

While frameworks and standardized processes are arguably critical to efforts toward responsible digital health, we should not let the clarity they provide obscure the complexity of the issues involved. For example, in “From General Principles to Procedural Values: Responsible Digital Health Meets Public Health Ethics, Nyrup proposes a move away from “principlist” approaches to a procedural approach, as modeled by the “accountability for reasonableness” (A4R) approach that has been influential in public health ethics. Nyrup argues that procedural approaches can overcome some of the commonly pointed out limitations of principlist approaches, for example, by highlighting rather than masking disagreements and by providing guidance on how to resolve trade-offs between different competing values.

Furthermore, in the paper by Roossien et al. (2021) mentioned above, the authors lead with an acknowledgment that the ethical implications of workplace health represent “a neglected topic and such a complex field of study that we cannot come up with solutions easily or quickly.” Their study is presented, not as an answer, but as a call for discussion. They also demonstrate a context-specific approach to investigating the ethics of workplace health interventions and argue that values such as privacy and autonomy cannot be isolated from other contextual elements as there is an inescapable “interplay between these values, the work context, and the responsibilities of workers and employers.”

## Conclusion

The work collected for this Research Topic presents current research insights, methods, tools, and examples of best practices that can inform responsible innovation and ethical practice in the design of digital health. It shows that while we are far from completely understanding how to responsibly design digital health services and technologies, we have an active and multidisciplinary community that can work together to advance knowledge on a responsible and sustainable future for our health and healthcare systems. To that end, we call upon researchers to engage in active discussion to enhance the diversity of views in digital health (both among researchers and those represented through research). Additionally, the complexity of researching ethics in digital health suggests there is a need for effective collaboration across disciplines to bring plurality to research and practice. The diversity of disciplines represented in this collection, ranging from design, human-computer interaction, philosophy, medicine and more, demonstrates a promising potential. We hope to see more collaborations across disciplines in the future.

## Author Contributions

DP wrote the outline of the editorial with KV, NA, and GL contributing for the papers they were responsible editor for. All authors discussed the contribution of the editorial and NA wrote the conclusion. All authors reviewed and edited the editorial.

## Conflict of Interest

The authors declare that the research was conducted in the absence of any commercial or financial relationships that could be construed as a potential conflict of interest.

## Publisher's Note

All claims expressed in this article are solely those of the authors and do not necessarily represent those of their affiliated organizations, or those of the publisher, the editors and the reviewers. Any product that may be evaluated in this article, or claim that may be made by its manufacturer, is not guaranteed or endorsed by the publisher.

